# Craniotabes in Newborns and the Role of Maternal Vitamin D Deficiency: A Case Series

**DOI:** 10.7759/cureus.73730

**Published:** 2024-11-15

**Authors:** Rita M Pita, Marta O Martins, Ana Ferraz, Raquel Henriques

**Affiliations:** 1 Neonatology Department, Maternidade Daniel de Matos, Unidade Local de Saúde de Coimbra, Coimbra, PRT

**Keywords:** craniotabes, neonate, newborn examination, pediatrics, vitamin d deficiency

## Abstract

Craniotabes is characterized by the softening of skull bones in newborns. It can be associated with conditions like rickets, congenital syphilis, and osteogenesis imperfecta. In otherwise healthy newborns, craniotabes is often linked to in utero vitamin D deficiency.

We report four cases of term infants diagnosed with craniotabes at birth. For all cases, laboratory tests confirmed vitamin D deficiency with serum 25-hydroxyvitamin D levels below 20 ng/mL, alongside maternal vitamin D deficiency. While three cases showed no complications, one newborn had a skull fracture and a neonatal stroke. All infants were supplemented with oral cholecalciferol, resulting in the normalization of serum 25-hydroxyvitamin D levels and resolution of the skull softening. These cases highlight the critical role of maternal vitamin D levels in fetal bone development and the potential risks of its deficiency. Although craniotabes associated with maternal vitamin D deficiency is a known condition among neonatologists, these cases help to increase awareness of this diagnosis. We highlight the need for thorough clinical examination, along with complementary diagnostic tests, to identify any related complications.

## Introduction

Craniotabes is characterized by the softening of the skull bones. When minimal pressure is applied by the examiner´s finger, an inward collapse of the skull is formed, followed by a rebound effect upon release [1]. This is described as the “ping-pong ball” effect.

The incidence of craniotabes in newborns ranges from 20% to 30% [1, 2]. It can be associated with conditions such as rickets, osteogenesis imperfecta, hypervitaminosis A, and congenital syphilis [1, 3]. More recently, craniotabes has also been linked to in utero vitamin D deficiency [1,4,5].

Vitamin D status in infants and children is defined based on serum 25-hydroxyvitamin D (25[OH]D) levels as deficiency (<20 ng/mL or <50 nmol/L), insufficiency (20-29 ng/mL or 50-74 nmol/L), and sufficiency (30-100 ng/mL or 75-250 nmol/L) [6].

In this case series, we report cases of four term infants diagnosed with craniotabes attributed to vitamin D deficiency.

## Case presentation

Case one

**Figure 1:**
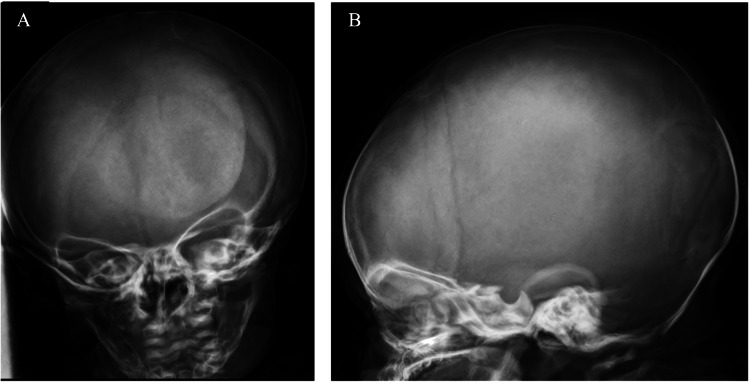
Normal skull X-ray with no evidence of fracture (A: anteroposterior view; B: lateral view)

A female infant was born at 39 weeks by vaginal delivery after an uneventful pregnancy, with birth weight adequate for gestational age (2840 g, 19^th^ percentile according to Fenton's growth chart). At birth, a “ping-pong ball” softening of the skull bones was noted on examination in the right temporal-occipital area. The infant appeared well, with a normal neurological examination and no other congenital or skeletal abnormalities. Her family history was unremarkable. No fractures were noted on the X-ray imaging of the skull (Figure 1). Laboratory investigation in the infant revealed a low 25(OH)D level, a low phosphorus level, and normal calcium. Maternal laboratory tests showed a low 25(OH)D level.

The infant was supplemented with oral vitamin D (2000 international units (IU)/day) [7,8], alongside her mother. She was regularly monitored at hospital follow-up appointments, showing normal growth and psychomotor development. Her cranial bones hardened within one month of birth. By the age of one year, she was discharged from hospital appointments.

Case two

**Figure 2:**
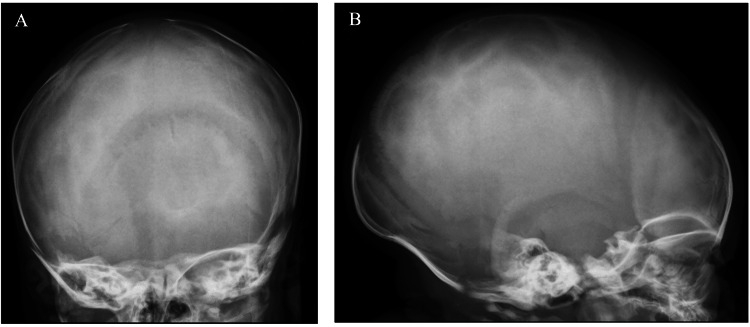
Normal skull X-ray with no evidence of fracture (A: anteroposterior view; B: lateral view)

A male infant was born at 40 weeks via vaginal delivery after an uneventful pregnancy, with a birth weight of 3330 g (30^th^ percentile, according to Fenton's growth chart). On the third day of life, a softening of the skull bones in the right parietal region was noted on physical examination. No abnormality was noted on the skull X-ray (Figure 2). Laboratory tests revealed low serum 25(OH)D level and increased parathyroid hormone (PTH) level. Serum calcium, phosphorus, and alkaline phosphatase (ALP) levels were within normal limits for the age. Further investigation revealed the serum 25(OH)D level of the mother was low. No other relevant family history was found.

The infant was supplemented with 2000 IU/day of oral vitamin D [7, 8] until laboratory reevaluation. The mother also started vitamin D supplementation upon the infant’s diagnosis.

Craniotabes resolved by the age of two months. He maintained follow-up until the age of five months, showing normal growth and psychomotor development. Laboratory reassessment at the age of five months revealed serum 25(OH)D and PTH levels within normal limits. The patient was discharged with instructions to maintain oral vitamin D at a dose of 400 IU/day until the age of one.

Case three

**Figure 3:**
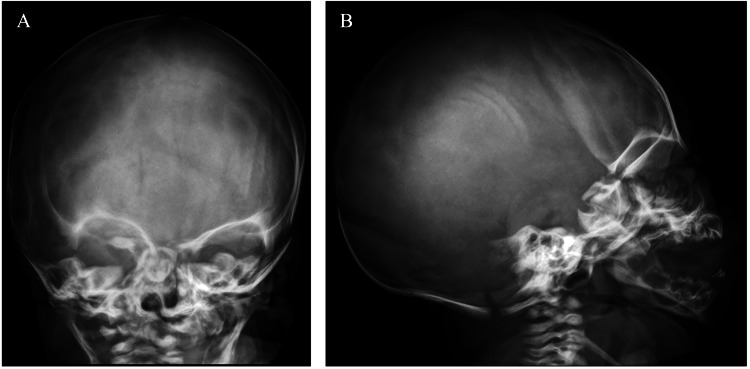
Normal skull X-ray with no evidence of fracture (A: anteroposterior view; B: lateral view)

A male infant was born at 38 weeks via vaginal delivery after an uneventful pregnancy, with a birth weight of 2710 g (16^th^ percentile, according to Fenton's growth chart). On examination at birth, a softening of the left parietal skull bone was noted. He also had a neonatal tooth. No other abnormalities were noted on physical examination. The skull X-ray was normal (Figure 3), and laboratory tests showed a low serum 25(OH)D level and normal serum calcium, phosphorus, ALP, and PTH levels.

The mother had insufficient levels of serum 25(OH)D, and there was no other relevant family history. The infant was started on oral vitamin D supplementation (2000 IU/day) [7, 8]. The mother was also supplemented with vitamin D.

At the age of one month, the skull examination was normal. Follow-up at four months showed normal growth and psychomotor development and a normal serum 25(OH)D level. The oral vitamin D was reduced to 400 IU/day, and no further follow-up was maintained.

Case four

A female infant was born at 38 weeks via vaginal delivery after an uneventful pregnancy. Her birth weight was 2690 g (21^st^ percentile, according to Fenton's growth chart), and the Apgar scores at one and five minutes were nine and 10, respectively.

Immediate examination after birth revealed a “ping-pong ball” effect and crepitus during the palpation of the right parietal skull region. The skull x-ray revealed a right parietal fracture. Laboratory tests showed vitamin D deficiency and increased PTH levels and ALP. Serum calcium was normal, and the phosphorus level was slightly decreased. The infant was started on oral vitamin D treatment (2000 IU/day) [7, 8]. The mother also had a low serum 25(OH)D level and started vitamin D supplementation.

**Figure 4:**
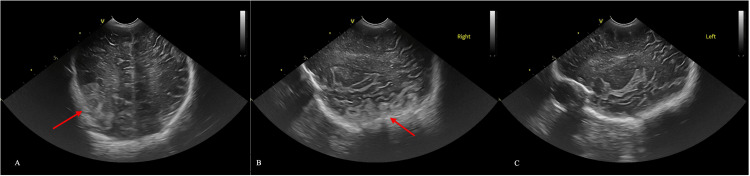
Cranial ultrasound images showing right parietotemporal hyperechogenicity in coronal view (A) and right parasagittal view (B); normal ultrasound images in left parasagittal view (C)

By the fourth day of life, widening of the coronal and lambdoid sutures was found. Cranial ultrasound revealed right parietotemporal hyperechogenicity, raising suspicions of right parietal hemorrhage/infarction (Figure 4).

**Figure 5:**
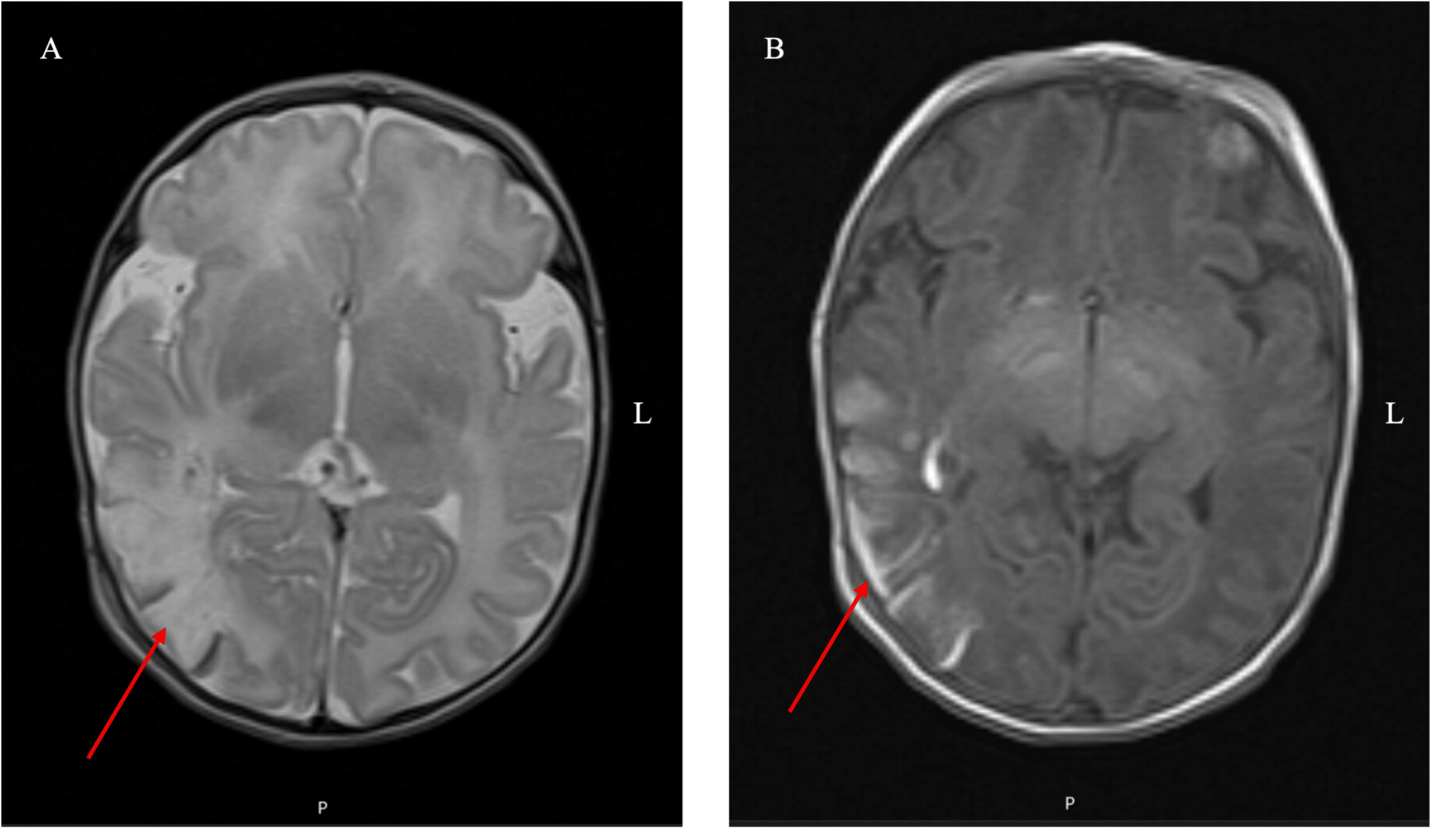
A) Brain MRI (T2-weighted imaging) showing loss of gray/white matter differentiation with abnormally high signal intensity involving the right temporal and parietal lobes in the territory of the right middle cerebral artery; B) hypersignal in the T1 sequence due to the presence of intracellular methemoglobin suggesting cortical hematic suffusion

Brain magnetic resonance imaging (MRI) confirmed an ischemic stroke with hemorrhagic transformation in the territory of the right middle cerebral artery (Figure 5). The electroencephalogram had no paroxysmal activity. The infant remained hospitalized for 10 days, with good clinical progress and without seizures.

Follow-up appointments during her first nine months of life revealed normal skull, growth, and psychomotor development. She had no clinical seizures or motor deficits. By four months of age, skull X-rays and laboratory reassessments confirmed the resolution of previous changes. Additionally, cranial ultrasounds at two and five months were unremarkable, showing no progression of the hyperechogenic area to porencephalic cysts. Thrombophilia and hemorrhagic study (prothrombin, activated partial thromboplastin and thrombin times; plasma concentration of fibrinogen, coagulation factors, antithrombin III, protein C and S; lupus anticoagulants; and molecular study of factor V Leiden, prothrombin, methylenetetrahydrofolate reductase, and plasminogen activator inhibitor-1 genes) were within normal limits. She maintains follow-up in outpatient neonatology and neuropediatrics appointments.

What do these cases have in common?

We report four cases of craniotabes resulting from maternal vitamin D deficiency. The main clinical characteristics at presentation are summarized in Table 1.

**Table 1:** Main characteristics of the four cases of craniotabes noted at presentation 25(OH)D: 25-hydroxyvitamin D; ALP: alkaline phosphatase; D: days; n.a. : not available; PTH: parathyroid hormone; W: weeks

	Case 1	Case 2	Case 3	Case 4	Reference values
Sex	Female	Male	Male	Female	n.a.
Gestational age	39w+4d	40w+3d	38w+5d	38w+3d	n.a.
Type of delivery	Vaginal	Vaginal	Vaginal	Vaginal	n.a.
Craniotabes location	Right temporal-occipital	Right parietal	Left parietal	Right parietal	n.a.
Skull x-ray	Normal	Normal	Normal	Right parietal fracture	n.a.
25(OH)D (ng/mL)	11.0	10.0	15.0	<7.0	Deficiency: < 20 ng/mL; Insufficiency: 20-29 ng/mL; Sufficiency: 30-100 ng/mL
Calcium (mg/dL)	9.6	9.4	9.7	8.8	7.8-10.6 mg/dL
Phosphorus (mg/dL)	5.2	7.1	6.4	5.3	5.5-10.5 mg/dL
PTH (pg/mL)	n.a.	125	63	142	6.4-88.5 pg/mL
ALP (U/L)	n.a.	125	211	297	90-273 U/L
Mother’s 25(OH)D (ng/mL)	11	19	29	11	Deficiency: < 20 ng/mL; Insufficiency: 20-29 ng/mL; Sufficiency: 30-100 ng/mL
Associated complications	None	None	None	Neonatal stroke (right middle cerebral artery)	n.a.

In all cases, skull softening was detected during the first three days after birth, primarily in the parietal region (n = 3). All neonates were born by vaginal delivery and adapted well to extrauterine life. A skull x-ray was performed in all cases, only identifying a skull fracture in case four. This infant was later diagnosed with an ischemic stroke in the territory of the right middle cerebral artery, the same side of the craniotabes. The remaining cases had no associated complications. All infants and mothers started with oral vitamin D supplementation immediately after diagnosis. During follow-up, skull softening resolved in the first two months of life for all cases, with no further complications. Growth and psychomotor development were normal for all infants during follow-up.

## Discussion

The reported cases contribute to further understanding of the relationship between craniotabes and vitamin D deficiency, highlighting the crucial role of vitamin D in neonates. Vitamin D plays an essential role in bone development and health, primarily by enhancing the intestinal absorption of calcium and phosphorus through its active metabolite, 1,25-dihydroxyvitamin D (1,25[OH]2D). It also supports neuromuscular and cardiac function and bone remodeling [9,10]. In both the fetus and newborn, vitamin D levels depend on maternal status, with studies showing that umbilical cord blood 25(OH)D levels are 60%-85% of the maternal values [11,12]. This maternal transfer is especially relevant in the third trimester when most fetal mineral accumulation occurs [11]. Therefore, maternal vitamin D deficiency can lead to fetal hypovitaminosis D and increase the risk of bone disorders.

Low vitamin D levels reduce calcium and phosphorus absorption, leading to decreased serum ionized calcium that triggers an increase in PTH production. This rise promotes calcium reabsorption in the kidneys, 1,25(OH)2D production, and osteoclastic bone reabsorption, releasing calcium into the bloodstream. An ALP increase might also be present [9]. As a result, maternal vitamin D deficiency during pregnancy can compromise fetal bone mineralization, manifesting as craniotabes, a softening of the skull bones, typically seen in the parietal region [1]. This condition is characterized by an inward collapse of the skull when pressure is applied, followed by a rebound, known as the “ping-pong ball” effect. Craniotabes may affect up to one-third of newborns [1,2], although case reports remain scarce [4,5,13]. Craniotabes may be a subtle manifestation and thereby may be underdiagnosed.

All reported cases demonstrated vitamin D deficiency through lab tests and were promptly started on cholecalciferol (vitamin D3) supplementation with doses of 2000 IU/day until they reached 25(OH)D sufficiency. Follow-up confirmed the resolution of the skull softening within the first two months of life and vitamin D sufficiency levels between the ages of four and five months. There is no consensus in the literature regarding the dose and duration of vitamin D3 replacement therapy in term infants with vitamin D deficiency. The global consensus on nutritional rickets recommends the administration of 2000 IU/day of vitamin D in patients under the age of one for at least 12 weeks if nutritional rickets was found. However, no recommendations are given for vitamin D deficiency without rickets [7]. The 2023 Polish guidelines suggest 2000 IU/day with follow-up testing in four to six weeks for vitamin D deficiency or rickets [8]. The Royal Osteoporosis Society recommends 3000 IU/day for eight to 12 weeks in infants aged one to five months with hypovitaminosis D [14].

Once vitamin D sufficiency was reached, daily doses were reduced to the standard 400 IU/day, following European Society for Paediatric Gastroenterology, Hepatology, and Nutrition (ESPGHAN) guidelines [15].

Craniotabes is generally considered a benign condition that resolves spontaneously within the first two or three months of life and typically without complications [16]. In this study, resolution occurred in the first two months of life for all cases. However, one infant developed a skull fracture and an asymptomatic ischemic stroke in the middle cerebral artery territory, which was suspected due to widened sutures observed during the examination. To our knowledge, there are no previously reported cases of neurological complications associated with craniotabes. Although a fracture may occur in a non-instrumented vaginal delivery, this is a rare event. A possible explanation for the relationship between craniotabes and the ipsilateral ischemic stroke may be the cranial compression of the softened area, causing compression and subsequent infarction.

Regarding maternal vitamin D deficiency, routine 25(OH)D testing during pregnancy is not a standard practice in Portugal. Maternal serum 25(OH)D levels ranged from 11 to 29 ng/mL in the reported cases, below the sufficiency level of 30 ng/mL suggested for adults [16]. Mothers diagnosed with vitamin D deficiency, particularly those breastfeeding, should begin supplementation to support both maternal and infant health.

## Conclusions

The reported cases of craniotabes due to in utero vitamin D deficiency provide important insights into the connection between maternal vitamin D levels and neonatal health. Routine screening for craniotabes in newborns is essential for early diagnosis and timely intervention. Identifying and addressing vitamin D deficiency in pregnant women is crucial in preventing this condition and safeguarding fetal bone development. We highlight the need for thorough clinical examination, along with complementary diagnostic tests, to identify any related complications.
